# Improvements on the Interfacial Properties of High-k/Ge MIS Structures by Inserting a La_2_O_3_ Passivation Layer

**DOI:** 10.3390/ma11112333

**Published:** 2018-11-20

**Authors:** Lu Zhao, Hongxia Liu, Xing Wang, Yongte Wang, Shulong Wang

**Affiliations:** Key Laboratory for Wide Band Gap Semiconductor Materials and Devices of Education, School of Microelectronics, Xidian University, Xi’an 710071, China; lzhaoxd@163.com (L.Z.); mikewyt@163.com (Y.W.); slwang@xidian.edu.cn (S.W.)

**Keywords:** atomic layer deposition, Ge-based MIS, surface passivation, interfacial properties

## Abstract

In this paper, the impact of La_2_O_3_ passivation layers on the interfacial properties of Ge-based metal-insulator-semiconductor (MIS) structures was investigated. It was proven that the formation of a thermodynamically stable LaGeO*_x_* component by incorporating a La_2_O_3_ interlayer could effectively suppress desorption of the interfacial layer from GeO_2_ to volatile GeO. The suppression of GeO desorption contributed to the decrease in oxide trapped charges and interfacial traps in the bulk of the gate insulator, or the nearby interfacial regions in the Al_2_O_3_/La_2_O_3_/Ge structure. Consequently, the hysteretic behavior of the dual-swept capacitance-voltage (*C*-*V*) curves and the frequency dispersion of multi-frequency *C*-*V* curves were remarkably weakened. Besides, more than one order of magnitude decrease in the gate leakage current density, and higher insulator breakdown electric field were obtained after inserting a La_2_O_3_ passivation layer.

## 1. Introduction

For a very long time, the excellent properties of a SiO_2_/Si interface, with high band offsets and few interfacial defects, have been the key reasons for supporting Si use as the main semiconductor material in integrated circuits (IC) [1]. However, with the continued scaling of the device’s size feature on a Si-based complementary metal oxide semiconductor (CMOS) process, high dielectric constant (high-k) materials have been introduced as alternative gate oxide dielectrics to replace ultrathin SiO_2_. The purpose was to reduce gate leakage current and power consumption beyond 45-nm technology nodes [2,3]. The introduction of high-k dielectrics into the Si-based CMOS process results in poorer insulator/Si interfaces compared to that of SiO_2_/Si, bringing in some unfavorable impacts such as instability problems and mobility degradation [4]. Considering this, alternative technological progress to dimension scaling, such as changing channel material, is also necessary for achieving high performance devices [5]. Owing to its high intrinsic hole mobility, Ge has drawn remarkable attention for realizing high performance applications in the past decade [6,7]. Ge-based metal-insulator-semiconductor (MIS) devices have shown great potential for integration into the Si CMOS technology, since promising electrical characteristics beyond those of Si devices could be realized by high-k/Ge structures [8]. However, there are some obstacles to overcome before employing Ge into a CMOS-compatible processing scheme with high performance. One of the most critical issues to solve is the Ge surface passivation engineering prior to the deposition of gate oxides [9]. For most high-k dielectric films deposited on Ge substrates without any surface passivation treatments, the generation of unstable Ge oxides is unavoidable during the thermal annealing process. That is, at temperatures higher than 400 °C, GeO_2_ reacts with Ge atoms at high-k dielectrics/Ge interface, which then form substoichiometric Ge oxides, including volatile GeO [10,11]. The desorption of GeO brings in a large number of structural defects, which would deteriorate the properties of the insulator/Ge interface [12].

Recently, it has been reported that rare earth oxides (REOs; i.e., Y_2_O_3_, CeO_2_, Sm_2_O_3_, and La_2_O_3_) show high affinity for Ge atoms. That is, the strong reaction between REOs and Ge substrates leads to the catalytic oxidation of Ge, which results in the spontaneous formation of stable interfacial layers [13,14,15,16]. Amongst the REOs, due to their large dielectric constant and high band offset relative to Ge, La-based oxides are considered as one kind of promising alternative gate dielectrics in Ge-based MIS devices, which can achieve more aggressive equivalent oxide thickness (EOT) scaling [17,18]. Furthermore, as the interface between La-based oxides and Ge substrates shows much better thermodynamic stability than that of GeO_2_/Ge, La-based oxides have promising interfacial passivation effects and could improve the electrical performance of Ge-based MIS devices [19,20]. Considering this, the effects of inserting a La_2_O_3_ passivation layer between an Al_2_O_3_ dielectric and Ge substrate, on the interfacial properties of Al/Al_2_O_3_/Ge and Al/Al_2_O_3_/La_2_O_3_/Ge MIS structures was investigated in this paper.

## 2. Experimental Section

La_2_O_3_ and Al_2_O_3_ gate stack films were deposited on n-type Ge (100) substrates, with electrical resistivity of about 0.1–1 Ω·cm, in an atomic layer deposition (ALD) reactor (R-150, Picosun, Espoo, Finland). Prior to the deposition, Ge substrates were treated with acetone and hydrous alcohol, and then cyclically dipped into a diluted HF solution (HF:H_2_O = 1:50) 5 times to remove the native GeO*_x_* layer. During the deposition process, La(^i−^PrCp)_3_ and trimethylaluminum (TMA) were used as La and Al precursors, respectively, while H_2_O was used as an oxidant. The precursors were alternately introduced to the reactor chamber, and were carried by high purity N_2_ (>99.999%). A typical ALD growth cycle for La_2_O_3_ was 0.3 s La(^i−^PrCp)_3_ pulse, followed by 4 s N_2_ purge and 0.3 s H_2_O pulse, followed by 9 s N_2_ purge. The Al_2_O_3_ ALD cycle structure was set as 0.1 s TMA pulse/3 s N_2_ purge/0.1 s H_2_O pulse/4 s N_2_ purge. By varying the number of ALD cycles, a 2 nm La_2_O_3_ oxide layer was deposited on the cleaned Ge substrate at 300 °C, followed by deposition of a 4 nm Al_2_O_3_ layer. For comparison, a control sample with only 6 nm Al_2_O_3_ as gate dielectrics was also prepared. After the deposition, rapid thermal annealing (RTA) was performed at 600 °C for 90 s in N_2_ ambient for both Al_2_O_3_/La_2_O_3_/Ge and Al_2_O_3_/Ge structures.

The surface morphology of the deposited films was monitored using atomic force microscopy (AFM, Dimension 3100, Veeco Digital Instruments by Bruker, Billerica, MA, USA) in tapping mode. The physical thickness was optically measured using Woollam M2000D spectroscopic ellipsometry (SE, Woollam Co. Inc., Lincoln, NE, USA) fitted with a Cauchy model. The chemical bonding state of the samples related to Ge substrates was examined by X-ray photoelectron spectroscopy (XPS, Axis Ultra DLD, Kratos Analytical, Manchester, UK) measurements. MIS capacitor structures were used to evaluate the electrical properties of the deposited films. Al was evaporated using electron-beam evaporation as a metal gate through a shadow mask with a diameter of 300 μm followed by a post metallization annealing (PMA) carried out in 97% N_2_/3% H_2_ ambient at 400 °C for 20 min to form a good Ohmic contact with gate dielectrics. Then the electrical properties of the fabricated MIS capacitors were evaluated using an Agilent B1500A parameter analyzer (Santa Clara, CA, USA).

## 3. Results and Discussion

**Figure 1:**
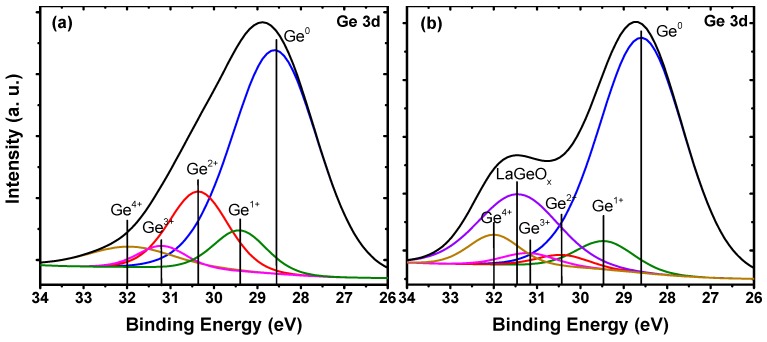
Shallow core-level spectra of Ge 3*d* for the (**a**) 6-nm Al_2_O_3_/Ge structure, and (**b**) 4-nm Al_2_O_3_/2-nm La_2_O_3_/Ge structure.

The Ge 3*d* spectra of the samples with and without the La_2_O_3_ passivation layer are shown in Figure 1. During the analysis of XPS data, the C 1*s* peak extracted from adventitious carbon at 284.6 eV was chosen as a bonding energy calibration reference. Compared with Figure 1a, a noteworthy change in Figure 1b was the appearance of a LaGeO*_x_* peak, indicating that a LaGeO*_x_* component was generated after inserting a thin La_2_O_3_ interlayer. Besides, the Ge oxide (GeO*_x_*) spectra could be divided into four Gaussian–Lorentzian line shape peaks (Ge^1+^, Ge^2+^, Ge^3+^, and Ge^4+^), which were located at a higher binding energy with respect to the Ge^0^ peak, with energy shifts of 0.8, 1.8, 2.6, and 3.4 eV, respectively [21]. The existence of these GeO*_x_* species was caused by the formation of an interfacial layer between gate dielectric films and Ge substrates [22]. Among the Ge sub-oxides, GeO (Ge^2+^) is known to adversely affect interfacial properties in contrast to other Ge oxides (Ge^1+^ and Ge^3+^), since GeO volatilization would cause a huge number of structural defects [13]. Compared with the control sample, a visible reduction in the intensity of the Ge^2+^ peak was observed in the Al_2_O_3_/La_2_O_3_/Ge case, indicating that to a certain extent, the formation of GeO was restrained by inserting a La_2_O_3_ passivation layer. Besides, it was observed that the intensity of the Ge^4+^ peak increased a bit after inserting a La_2_O_3_ passivation layer. Such variations in Ge^2+^ and Ge^4+^ peaks displayed a reasonable self-consistency, which was mainly ascribed to the reduction of desorption from GeO_2_ (Ge^4+^) to GeO, benefiting from the generation of a thermodynamically stable LaGeO*_x_* component near the La_2_O_3_/Ge interface [23].

**Figure 2:**
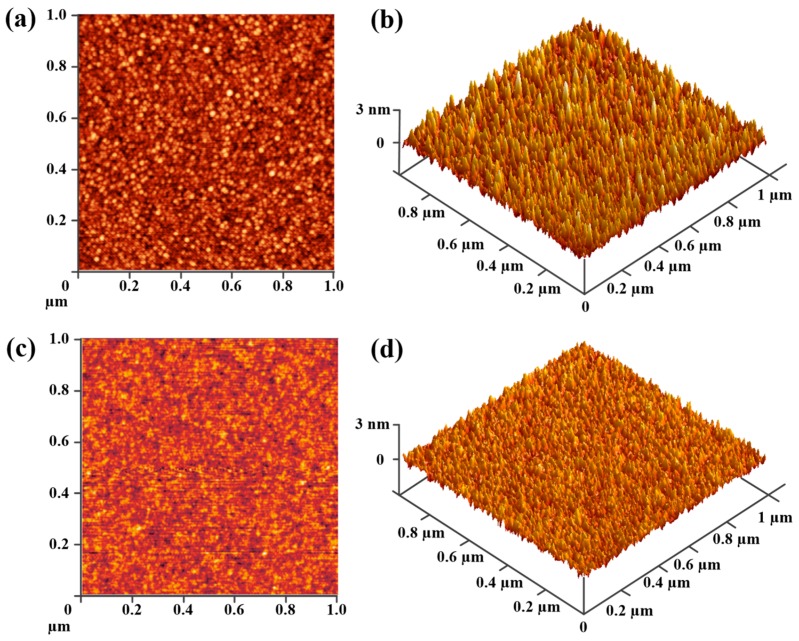
(**a**) Two- and (**b**) three-dimensional AFM images of the Al_2_O_3_ films on Ge substrates without La_2_O_3_ passivation; and (**c**) two- and (**d**) three-dimensional AFM images of the Al_2_O_3_ films on Ge substrates with a La_2_O_3_ interfacial passivation layer.

The two- and three-dimensional AFM images of the Al_2_O_3_ films deposited on Ge substrates with and without a La_2_O_3_ interlayer are shown in Figure 2. The scan size of each AFM image was 1 × 1 µm^2^. The root-mean-square (RMS) surface roughness of the films was extracted from the AFM images. The surface roughness of the sample without a La_2_O_3_ interlayer was about 0.62 nm in RMS, which was relatively a large RMS value for dielectric films deposited by ALD. We ascribed this large RMS value to the degradation of interfacial smoothness caused by the desorption of volatile GeO nearby the Al_2_O_3_/Ge interface during the high temperature post-deposition annealing (PDA) process [24]. While for the sample with a La_2_O_3_ interfacial passivation layer, an obviously smaller RMS value of 0.23 nm was observed. Such a reduction in the RMS value suggested that the desorption of GeO was suppressed since the existence of a thermodynamically stable LaGeO*_x_* layer between gate oxides and Ge substrates restrained the generation of volatile GeO. The decrease in volatile GeO had a positive impact on the electrical performance in the Al_2_O_3_/La_2_O_3_/Ge case. This improvement will be discussed in detail in the following capacitance-voltage (*C*-*V*) and gate leakage current density-voltage (*J*-*V*) parts.

**Figure 3:**
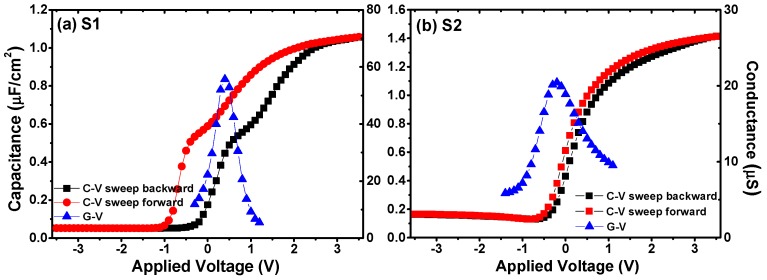
*C*-*V* characteristics for the fabricated MIS capacitors using Al_2_O_3_ films as insulators (**a**) without and (**b**) with a La_2_O_3_ interfacial passivation layer.

To further investigate the effects of a La_2_O_3_ interfacial passivation layer on the interfacial properties of Ge-based MIS structures, the electrical properties of the fabricated MIS capacitors were analyzed. Figure 3 shows the *C*-*V* and conductance-voltage (*G*-*V*) characteristics of the fabricated MIS capacitors using Al_2_O_3_ as an insulator, with and without a La_2_O_3_ passivation layer. For simplicity, the MIS capacitor using only Al_2_O_3_ as an insulator was marked as S1, and the MIS capacitor with La_2_O_3_ as an interfacial passivation layer was assigned as S2. The dual-swept *C*-*V* curves were obtained by biasing the gate-applied voltage from accumulation to inversion (backward sweep), and sweeping back (forward sweep) at 100 kHz. *G*-*V* measurements were performed simultaneously with the backward swept *C*-*V* curves. The capacitance values at the accumulation region of the *C*-*V* curves for capacitor S1 and S2 were 1.06 and 1.41 μF/cm^2^, respectively. The dielectric constant values of the samples were estimated using the following equations [25,26]:

(1) $$C_{\mathrm{ox}}=C_{\mathrm{ac}}\left[1+{\left(\frac{G_{\mathrm{ac}}}{\omega C_{\mathrm{ac}}}\right)}^{2}\right]$$

(2) $$CET=\frac{\varepsilon_{0}\varepsilon_{\mathrm{Si}O_{2}}A}{C_{\mathrm{ox}}}$$

(3) $$k=\frac{\varepsilon_{\mathrm{Si}O_{2}}t_{\mathrm{ox}}}{CET}$$

where *C*_ac_ is the capacitance value at the accumulation region, *G*_ac_ is the conductance corresponding to the accumulation region of the *C*-*V* curves, *ω* is the angular frequency, *C*_ox_ is the oxide capacitance of dielectric films, *A* is the electrode area, *t*_ox_ is the measured thickness of gate dielectrics, $\varepsilon_{0}$ and $\varepsilon_{\mathrm{Si}O_{2}}$ are the permittivity values of vacuum and SiO_2_, respectively. The physical thickness of the gate dielectric films in S1 and S2 was measured to be 6.42 and 6.83 nm, separately. Therefore, the effective dielectric constant values of S1 and S2 were calculated to be 7.95 and 11.10, respectively. The increment in the dielectric constant value for the sample with a La_2_O_3_ interfacial passivation layer was attributed to the introduction of La elements into dielectric films, since the *k* values of La_2_O_3_ [27], and La_x_Al_y_O [28] formed by the interdiffusion of La_2_O_3_ and Al_2_O_3_ were much higher than that of Al_2_O_3_ [29].

The flat band voltages (*V*_FB_) of the *C*-*V* curves in Figure 3 were extracted from the Hauser NCSU CVC simulation software (North Carolina State University, Raleigh, NC, USA), taking quantum mechanical effects into account [30]. It was observed that the Al/Al_2_O_3_/Ge MIS capacitor showed a much larger flat band voltage hysteresis width (Δ*V*_FB_), ∼863 mV, than that of S2 (∼174 mV). The hysteretic behavior of the dual-swept *C*-*V* curves has been proven to be caused by the existence of oxide trapped charges (*Q*_ot_) in the bulk of the gate insulator or nearby the interfacial region [31]. Using the midgap charge separation method, the oxide-trapped charge density (*N*_ot_) for the fabricated capacitors was calculated using the following equation [32]:

(4) $$N_{\mathrm{ot}}=\frac{\Delta V_{\mathrm{FB}}C_{\mathrm{ox}}}{qA}$$

where Δ*V*_FB_ is the hysteresis width of *V*_FB_, *C*_ox_ is the oxide capacitance, *q* is the elementary charge (1.602 × 10^−19^ C), and *A* is the electrode area. The *N*_ot_ values were calculated to be 5.91 × 10^12^ cm^−2^ for the Al/Al_2_O_3_/Ge MIS capacitor, and 1.56 × 10^12^ cm^−2^ for the case with a La_2_O_3_ passivation layer. A visible decrease of *N*_ot_ was observed after inserting a La_2_O_3_ passivation layer.

**Figure 4:**
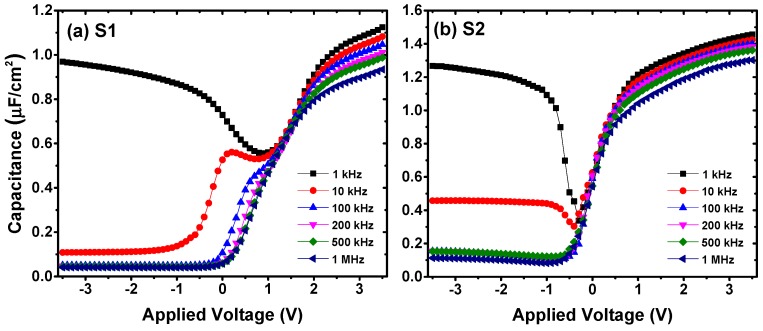
*C*-*V* characteristics measured at various frequencies for the fabricated MIS capacitors using Al_2_O_3_ films as insulators (**a**) without, and (**b**) with a La_2_O_3_ interfacial passivation layer.

The *C*-*V* curves of the MIS capacitors without the La_2_O_3_ interlayer showed a significant anomalous hump phenomena at the weak inversion regions as shown in Figure 3a, which were reported to be caused by slow interfacial traps existing between the gate insulator and substrate [33]. Besides, as shown in Figure 4, different amounts of interfacial traps caused different degrees of frequency dispersion phenomena at the weak inversion regions of the *C*-*V* curves measured at multi-frequencies. Considering this, we discussed the interface state density (*D*_it_) values of S1 and S2, extracted from the combination of the backward swept *C*-*V* and *G*-*V* characteristics, using the following relation of single-frequency approximation method [29,34]:

(5) $$D_{\mathrm{it}}=\frac{2}{qA}\frac{\frac{G_{\max}}{\omega }}{\left[{\left(\frac{G_{\max}}{\omega C_{\mathrm{ox}}}\right)}^{2}+{\left(1-\frac{C_{\max}}{C_{\mathrm{ox}}}\right)}^{2}\right]}$$

where *A* is the electrode area, *C*_ox_ is the gate oxide capacitance as defined in Equation (1), *G*_max_ is the peak value of *G*-*V* curve, and *C*_max_ is the capacitance corresponding to *G*_max_ at the same gate-applied voltage. The various parameter results discussed above are shown in Table 1. For the fabricated MIS capacitors without a La_2_O_3_ passivation layer, the value of *D*_it_ was about 1.13 × 10^13^ eV^−1^·cm^−2^. After the Ge surface passivation treatment using a La_2_O_3_ interlayer, the *D*_it_ value decreased evidently to ∼4.97 × 10^12^ eV^−1^·cm^−2^, indicating that to some extent, the insertion of a La_2_O_3_ passivation layer inhibited the generation of interface traps, which gave an explanation to the pronounced weak anomalous hump tendency. It is worth noting that, the frequency dispersion phenomenon was also observed at the accumulation region of the *C*-*V* curves. It has been reported by Kouda et al. that the frequency-dependent variations in the dielectric constant of the gate stacks caused by oxygen vacancies should be responsible for the frequency dispersion phenomenon at the accumulation region [14,35]. As shown in Figure 4, the frequency dispersion phenomenon at the accumulation region became weaker after inserting a La_2_O_3_ interlayer, indicating that the introduction of this interlayer suppressed the generation of oxygen vacancies in the stack structures.

The contribution of the La_2_O_3_ interfacial passivation layer to the decrease in trapped oxide charges and interfacial traps could be explained as follows; when Al_2_O_3_ dielectrics were deposited on the Ge substrates without any passivation treatment, the outdiffusion of Ge atoms to the Al_2_O_3_ dielectric films generated unstable Ge oxides at the Al_2_O_3_/Ge interface. The quality of the interface between Ge and its oxides tends to deteriorate during the PDA process because the decomposition or desorption from GeO_2_ to GeO following the reaction equation of GeO_2_ + Ge → 2GeO leads to the formation of structural defects mainly consisting of dangling bonds and oxygen vacancies [36]. While after inserting the La_2_O_3_ interfacial passivation layer, La_2_O_3_ could react with the outdiffused Ge atoms to form the stable LaGeO*_x_* compound, which would effectively suppress the generation of volatile GeO, contributing to the decrease of structural defects in the bulk of the insulator and/or in the interfacial region. As a result, the *N*_ot_ and *D*_it_ values were obviously decreased by inserting a La_2_O_3_ passivation layer of insulator/Ge interfaces, contributing to the suppression of hysteresis in dual-swept *C*-*V* curves and frequency dispersion in multi-frequency *C*-*V* curves.

**Figure 5:**
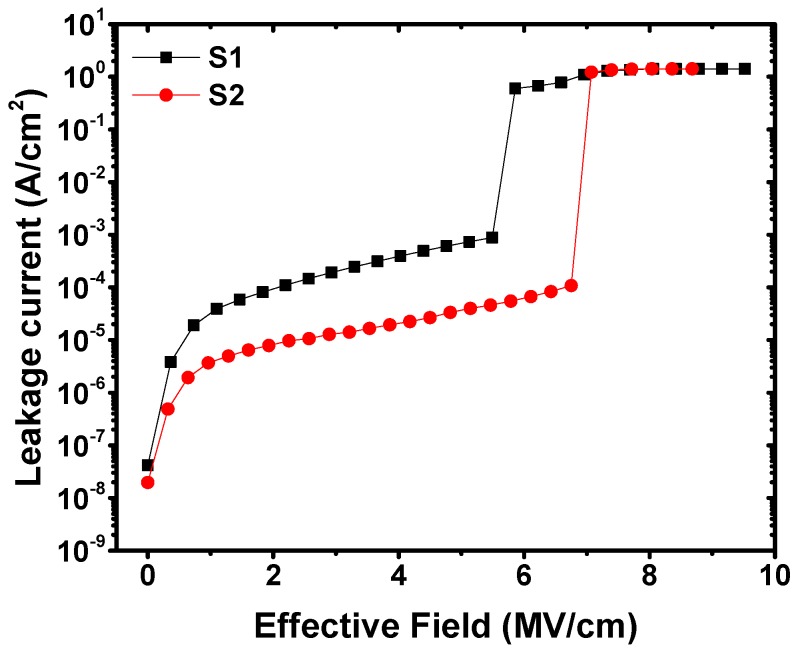
*J*-*V* characteristics for fabricated MIS capacitors using Al_2_O_3_ films as insulators without and with a La_2_O_3_ interfacial passivation layer.

Figure 5 shows the gate leakage current density of the fabricated MIS capacitors as a function of the gate-applied electrical field. The gate leakage current density for capacitors S1 and S2 were measured to be 1.92 × 10^−4^ and 1.29 × 10^−5^ A/cm^2^ separately, when the gate-applied electrical field was 3 MV/cm. More than one order of magnitude decrease in the gate leakage current density was achieved after inserting a La_2_O_3_ passivation layer. Furthermore, it is worth noting that the gate insulator breakdown electric field of capacitor S2 (~7.07 MV/cm) was apparently higher than that of capacitor S1 (~5.86 MV/cm), which revealed that the inserted La_2_O_3_ passivation layer had a positive effect on the breakdown characteristics of the gate insulators. The improvements in gate leakage current density and gate insulator breakdown characteristics were suspected to benefit from the reduction of structural defects including dangling bonds and oxygen vacancies [37]. For the sample with a La_2_O_3_ passivation layer, less structural defects in the gate insulator meant a smaller possibility of creating a continuous chain connecting the gate electrode to the substrate semiconductor, contributing to the realization of lower gate leakage current density and higher insulator breakdown electric field [38].

## 4. Conclusions

The Ge surface engineering using La_2_O_3_ as a passivation layer was carried out and investigated systematically in this paper. The formation of a thermodynamically stable LaGeO*_x_* interfacial layer effectively suppressed the desorption of volatile GeO, resulting in smaller *N*_ot_ and *D*_it_ values achieved after the insertion of a La_2_O_3_ passivation layer compared with the control sample. These improvements on the interfacial properties significantly weakened the hysteresis in dual-swept *C*-*V* curves and frequency dispersion in multi-frequency *C*-*V* curves. Besides, the gate leakage current and insulator breakdown characteristics for the MIS structure with La_2_O_3_ passivation were also improved.

## Figures and Tables

**Table 1:** The electrical parameters extracted from the fabricated MIS capacitors without and with a La_2_O_3_ interfacial passivation layer.

Sample	*C*_ox_ (µF/cm^2^)	k	Δ*V*_FB_ (mV)	*N*_ot_ (cm^−2^)	*D*_it_ (eV^−1^·cm^−2^)
S1	1.096	7.95	863	5.91 × 10^12^	1.13 × 10^13^
S2	1.438	11.10	174	1.56 × 10^12^	4.97 × 10^12^
